# Protocol for immunofluorescence detection and quantitative analysis of pH-dependent transcriptional condensates

**DOI:** 10.1016/j.xpro.2026.104425

**Published:** 2026-03-11

**Authors:** Zhongyang Wu, Krishnan Raghunathan, Zhe Zhong, Jay Thiagarajah, Xu Zhou

**Affiliations:** 1 Division of Gastroenterology, Hepatology and Nutrition, Department of Pediatrics, Boston Children’s Hospital and Harvard Medical School, Boston, MA 02115, USA; 2 Broad Institute of Massachusetts Institute of Technology and Harvard, Cambridge, MA 02142, USA

**Keywords:** Cell culture, Immunology, Microscopy

## Abstract

Acidic pH regulates the assembly of transcriptional condensates containing BRD4 and MED1 in a variety of mouse and human cells. Here, we present a protocol to image and quantify BRD4 condensates in bone marrow-derived macrophages. We describe steps for preparing macrophage growth medium at controlled pH levels, performing immunofluorescence experiments, and acquiring images with Airyscan confocal and STED super-resolution microscopy. We detail image processing pipelines to analyze condensate properties using FIJI, CellProfiler, and a custom MATLAB program.

For complete details on the use and execution of this protocol, please refer to Wu et al.^1^

## Highlights

• Steps for pH adjustment of macrophage growth medium
• Instructions for pH treatment and immunofluorescence staining of BMDMs
• Details on the use of CellProfiler for condensates counting across pH conditions
• Details on the use of MATLAB for quantitative analysis of condensates properties

## Abstract (editor highlights)

Publisher’s note: Undertaking any experimental protocol requires adherence to local institutional guidelines for laboratory safety and ethics.

## Graphical abstract

**Figure:**
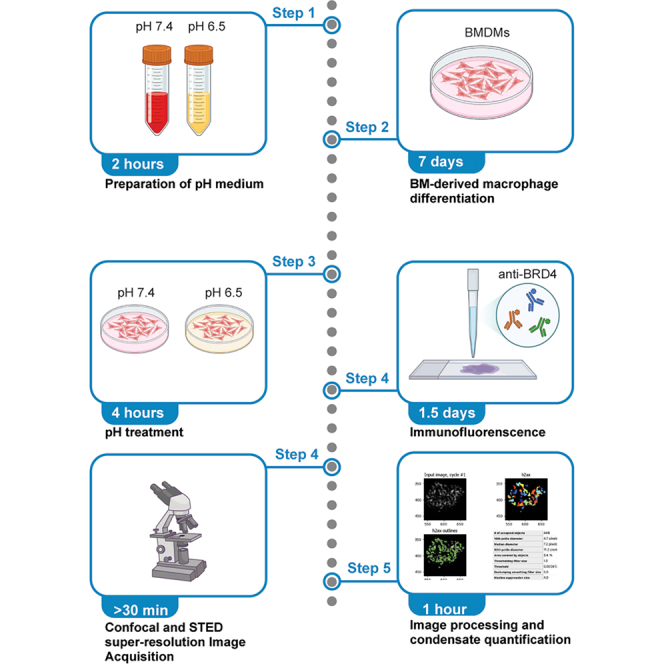

## Before you begin

This protocol describes the procedures for visualizing and quantifying BRD4 condensates in bone marrow-derived macrophages (BMDMs). It outlines medium pH adjustment, macrophage culture, immunofluorescence staining, and image analysis using confocal and STED microscopy. Although optimized for BMDMs, this protocol has been successfully adapted to other cell types, including fibroblasts, epithelial cells, T cells and B cells derived from either mouse or human origin. We expect that it can be applied to other cell types after calibration of medium pH, antibody concentration and proper acquisition settings.

BRD4 condensates are sensitive to pH. Cell culture medium recipe, supplements, culture duration, cultured cell types, specific cell treatment and incubator settings can all impact medium pH. Therefore, preparing and varying precise extracellular pH conditions is essential for reproducibility. The preparation of macrophage growth medium (MGM) at defined pH values (7.4, 6.5, and 6.0) and the equilibration of media under 5% CO_2_ are critical prerequisites before beginning cell treatment.

### Innovation

This protocol establishes a robust workflow to interrogate the pH-dependent behavior of BRD4 condensates in primary macrophages by integrating precise medium pH calibration, super-resolution STED microscopy. Quantitative analysis is automated through a custom MATLAB program that extracts condensate intensity, contour geometry, and cytosolic-nuclear distribution from raw STED images. The pipeline generates both raw datasets and visual summaries for transparent and reproducible quantification. By linking environmental pH shifts to immune responses, this protocol establishes a versatile framework for studying biophysical control of transcriptional condensates, readily adaptable to other nuclear factors, cell types, or stress conditions such as hypoxia or metabolic acidosis.

### Preparation of L929 conditioned medium

**Timing: 11–12 days**

1. Prepare complete L929 growth medium. In a sterile biosafety hood, per 500 mL standard RPMI base medium (Sigma Aldrich, Cat# R0883), supplement with the following reagents:
  a. Add 50 mL Fetal Bovine Serum (FBS).
  b. Add 5 mL L-Glutamine (200mM stock).
  c. Add 5 mL Sodium pyruvate (100mM stock).
  d. Add 5 mL penicillin-streptomycin (Pen/Strep).
  e. Add 5 mL HEPES, pH 7.4 (1M stock).

**Day 1**

2. Thaw L929 fibroblasts in 37°C water bath briefly and wash off the freezing medium with 10 mL pre-warmed L929 growth medium.
3. Seed 5 × 10^6^ in a T175 flask, with 35 mL L929 growth medium.
4. Grow cells at 37°C and 5% CO_2_ until they reach 100% confluency.

**Day 2**

5. When cells reach confluency, wash cells once with 15 mL1× PBS, and detach cells using 0.05% Trypsin.
6. Spin at 500× g for 5 min and collect the cell pellets.
7. Resuspend the cell pellets and reseed at 1.5 × 10^6^ L929 cells per T175 flask in 34 mL RPMI complete medium.

***Note:*** This represents approximately a 1:17 split ratio from the confluent medium. Cells are expected to reach confluence in 2 days.

**Figure 1:**
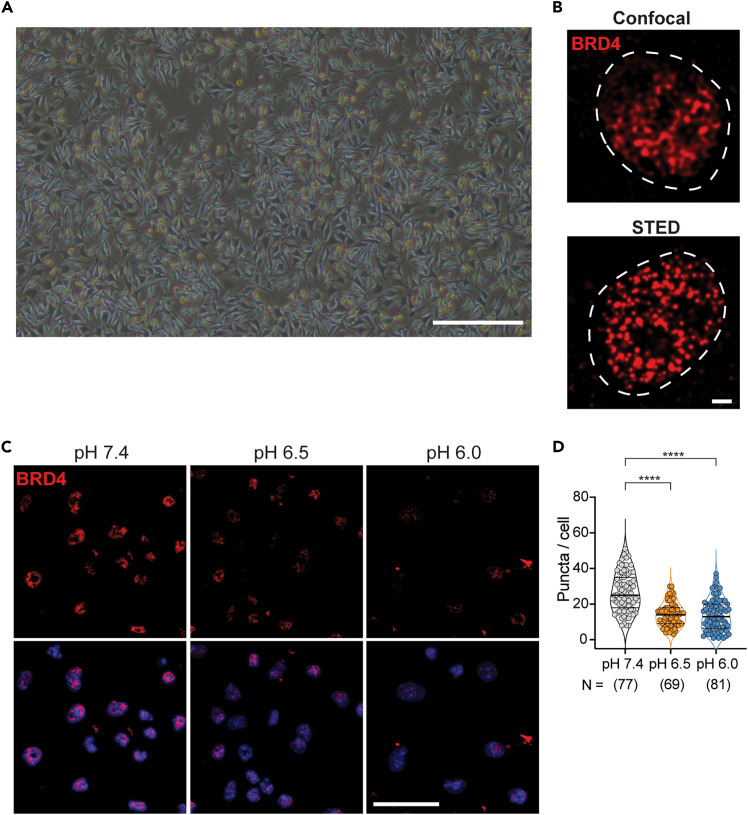
BRD4 condensates under acidic pH (A) Representative image of L929 mosaic confluency. Scale bar 100 μm. (B) Representative confocal and STED super-resolution images of BRD4 condensates. Scale bar 1 μm. (C) Immunofluorescence of BRD4 condensates in BMDMs cultured in medium at pH 7.4, 6.5, or 6.0 for 4 h. Scale bar 10 μm. (D) Quantification of BRD4 condensates in (C) using CellProfiler. ∗∗∗∗*p* < 0.0001.

***Note:*** At mosaic confluency stage, L929 cells display an uneven, patch-like growth pattern across the flask, with intermixed dense and sparse areas, rather than forming a smooth and uniform monolayer.

8. Monitor L929 cells daily. When cultures reach “mosaic confluency” (Figure 1A), incubate for an additional 10–11 days.

**Day 12**

9. Collect supernatant and filter with a 0.22 μm vacuum filtration unit to remove any cellular debris.
10. The filtered supernatant can be aliquoted and stored at −80°C for 6 months.

***Note:*** L929-conditioned medium contains macrophage colony-stimulating factor (M-CSF) produced from L929 cells. This is often used to support BMDM differentiation in place for recombinant M-CSF, at a reduced cost. Each batch of L929-conditioned should be tested for efficiency of BMDM differentiation.

**CRITICAL:** Use sterile technique throughout all steps to prevent contamination. Avoid extended culture periods as L929 viability and M-CSF secretion decline over time.

### Preparation of macrophage growth medium at varying pH

**Timing: 1.5 h**

11. Prepare complete macrophage growth medium (MGM).
  a. Prepare complete RPMI without sodium bicarbonate.
    In a sterile biosafety hood, per 500 mL RPMI base medium (without sodium bicarbonate, Cat# R7388 Sigma-Aldrich), supplement with the following reagents:
    i. Add 50 mL Fetal Bovine Serum (FBS).
    ii. Add 5 mL L-Glutamine (200mM stock).
    iii. Add 5 mL Sodium pyruvate (100mM stock).
    iv. Add 5 mL penicillin-streptomycin (Pen/Strep).
    v. Add 1.75 μL 2-Mercaptoethanol to final concentration 50μM.
    vi. Add 5 mL HEPES, pH 7.4 (1M stock).
      ***Note:*** The non-bicarbonate RPMI base medium contains 20 mM HEPES, so the final concentration of HEPES is ∼30 mM after supplementation. MGM can be prepared in advance and stored at 4°C for up to two weeks.
  b. Thaw L929-conditioned medium from −80°C.
  c. Add 30 ml L929-conditioned medium to 100 mL complete RPMI to generate MGM. Mix gently by inversion.
    ***Note:*** The L929-conditioned medium is prepared using complete RPMI containing 23.5 mM (2 g/L) NaHCO_3_. Thus, MGM contains approximately 5.4 mM NaHCO_3_ before pH adjustment. M-CSF levels may vary across batches depending on the L929 culture density and duration; therefore, the optimal volume of L929-conditioned medium may require adjustment with each preparation.
12. Adjust pH of MGM.
  a. pH 7.4: Bring the final NaHCO_3_ concentration to 30 mM to maintain pH at 7.4 in a 5% CO_2_ incubator. Then use 1N NaOH to adjust medium pH to 7.4. To measure pH, aliquot 3 mL of medium and incubate it in the CO_2_ incubator (37°C and 5% CO_2_) for at least 1h to equilibrate before measuring with a pH meter.
  b. pH 6.5: Use 1N HCl to adjust medium pH to 6.5. (As a reference, we add 1N HCl at ∼1:100 to make pH 6.5 MGM).
  c. pH 6.0: Use 1N HCl to adjust medium pH to 6.0. (As a reference, we add 1N HCl at 1:90 to make pH 6.0 MGM).

**CRITICAL:** Medium pH varies significantly depending on 5% CO2 equilibration; ensure the medium is equilibrated with 5% CO2 before measuring pH.

***Note:*** pH equilibration depends on CO_2_ concentration. To ensure reproducibility across experiments, equilibrate the medium in the same vessel used for cell culture (e.g., 15 cm dish) for at least 1 h at 37°C and 5% CO_2_ before final measurement. If pH adjusted media are stored for later use, verification of pH before experiment is recommended to ensure proper pH conditions. All the steps except pH measurement should be performed in a sterile cell culture hood. For pH measurement, take a small medium aliquot and avoid contaminating the bulk medium.

Extracellular pH ∼7.4 is thought as homeostatic physiological conditions, whereas mildly acidic pH (e.g., 6.0–6.8) mimics microenvironmental acidification that occur during inflammation, ischemia/hypoxia, and solid tumors. The selected pH range is intended to bracket physiologically relevant acid stress while maintaining BMDM viability. We found poor cell viability at pH range < 6.0.

### Preparation of bone-marrow-derived macrophages (BMDMs)

**Timing: 7 days**

13. Isolate bone marrow (BM) cells. The protocol for BM cells isolation is described in Gotaro Toda et al.,^2^
  a. Euthanize mouse wildtype C57BL/6 mice.
  b. Cut off the hind leg above the pelvic-hip joint and cut at the tibia ankle joint to dissect the tibia with sharp sterile scissors.
  c. Remove major muscles from femur and tibia.
  d. Remove the epiphyses of femur and tibia so that the bone marrow can be accessed from the ends with a 23G needle. Flush the bone marrow out onto a 70 μm nylon cell strainer placed in a 50 mL Falcon conical tube.
  e. Collect bone marrow and remove erythrocytes by incubating with ACK lysing buffer for 3 min.
  f. Resuspend the collected cells in 30 mL standard MGM and mix well.
  g. Day 0: plate BM cells per mouse in a 15 cm tissue-cultured dish with 30 mL MGM. Incubate overnight at 37°C and humidified 5% CO_2_ incubator.

***Note:*** The Initial step of plating cells in a tissue-cultured-treated dish allows removal of adherent cells (e.g., fibroblasts, differentiated macrophages), to enrich for BM-derived hematopoietic cells.

14. Differentiate BM cells into BMDMs.
  a. Day 1: Collect the floating BM cells and plate ∼1/5 mouse BM cells from an 8-weeks adult mouse in a non-tissue-culture-treated 15 cm dish containing 20 mL pre-warmed MGM.
  b. Maintain cells at 37°C, 5% CO_2_.
  c. Add 15 mL MGM on day 4 to support continuous growth and differentiation of BMDMs.
  d. Allow cells to differentiate into mature macrophages for a total of 6 to 7 days.

***Note:*** BM cells are small, round and floating cells. A small fraction of adherent macrophages is expected to appear around day 4 of differentiation. A significant increase of adherent macrophages is expected after refeeding with MGM. Mature BMDMs are expected to reach 2–3×10^7^ per 15 cm plate at Day 7.

### Preparation of gelatin-coated coverslips

**Timing: 20 min**

15. Coat coverslips with gelatin.
  a. Place glass coverslips in a 24-well plate.
  b. Sterilize the coverslips by adding 300 μL 75% ethanol per well and incubate for 30 s.
  c. Remove ethanol and allow coverslips to air-dry for 3–5 min.
  d. Add 300 μL 0.1% gelatin solution per well and incubate at 37°C for 15 min.
  e. Remove gelatin and keep the coated plates at RT or 4°C until use.

**CRITICAL:** All the steps must be performed in a sterile cell culture hood to avoid contamination.

## Key resources table

**Table:** 

REAGENT or RESOURCE	SOURCE	IDENTIFIER
**Antibodies**		

Rabbit monoclonal to BRD4 (IF and STED, 1:100 dilution)	Abcam	Cat# ab128874; RRID: AB_11145462
Abberior STAR Red, anti-rabbit (STED,1:250 dilution)	Abberior	Cat# STORANGE-1002-500UG; clone: N/A; RRID: AB_3068622
Rabbit (DA1E) Monoclonal Antibody IgG Isotype Control (IF, 1:200 dilution)	Cell Signaling Technology	Cat#3900; RRID: AB_1550038; clone: DA1E
Rabbit Alexa fluor 647 (IF, 1:1000 dilution)	Invitrogen	Cat# A21245

**Chemicals, peptides, and recombinant proteins**		

RPMI-1640 Medium	Sigma-Aldrich	Cat# R0883
RPMI-1640 without sodium bicarbonate	Sigma-Aldrich	Cat# R7388
Fetal bovine serum	R&D systems	Cat# S11595H
HEPES	Gibco	Cat# 15630080
L-glutamine	Gibco	Cat# 25030081
Sodium pyruvate	Gibco	Cat# 11360070
Penicillin/Streptomycin	Gibco	Cat# 15140122
Hoechst 33342 solution	Thermo Scientific	Cat# 62249
Hydrochloric acid solution	Sigma-Aldrich	Cat# H9892
Sodium hydroxide solution	Sigma-Aldrich	Cat# S2770
Paraformaldehyde 16% Aqueous Solution	Electron Microscopy Science	Cat# 15710
ProLong Glass Antifade Mountant	Invitrogen	Cat# P36984
Triton X-100	Sigma-Aldrich	Cat# X100–500ML
Fetal bovine serum	R&D systems	Cat# S11595H
2-Mercaptoethanol	Sigma-Aldrich	Cat# M3148
ACK lysis buffer	Gibco	Cat# A1049201
0.1% gelatin	Merck Millipore	Cat#ES-006-B
0.5% Trypsin-EDTA, no phenol red	Thermo Scientific	Cat#15400054
PBS pH 7.4 (1X)	Gibco	Cat# 10010-023

**Experimental models: Cell lines**		

BMDMs	This paper	N/A

**Software and algorithms**		

Cellprofiler	Stirling et al.^3^	https://cellprofiler.org/
Fiji/ImageJ version 2.0.0-rc-59/1.51n	Open source	https://imagej.net/software/fiji/
GraphPad Prism 9.0	GraphPad Software	N/A
Microsoft Excel	Microsoft	N/A
MATLAB®	MATHWORKS	R2023a
Bftools	The Open Microscopy Environment	N/A

## Materials and equipment

**Table:** 4% formaldehyde fixation solution (4% v/v)

Reagent	Final concentration	Amount
Formaldehyde	4%	1 mL
PBS without Ca^2+^ and Mg^2+^ Gibco, #10010-023	96%	3 mL
Total	N/A	4 mL

Prepare by diluting 16% formaldehyde stock 1:4 in PBS. Store at 4°C for up to 2 weeks.

***Note:*** Formaldehyde is toxic and a potential carcinogen. Prepare and handle 4% PFA in a certified chemical fume hood while wearing lab coat, nitrile gloves, and eye/face protection. Dispose of PFA-containing solution and contaminated materials as hazardous chemical waste according to institutional guidelines.

**Table:** Permeabilization buffer (0.5% Triton X-100 in PBS)

Reagent	Final concentration	Amount
Triton X-100	0.5%	250 μL
PBS without Ca^2+^ and Mg^2+^ Gibco, #10010-023	99.5%	49.75 mL
Total	N/A	50 mL

Mix thoroughly until Triton X-100 is fully dissolved. Store 4°C or room temperature for up to 1 month.

**Table:** Blocking buffer (5% BSA, 0.3% Triton X-100 in PBS)

Reagent	Final concentration	Amount
BSA	5%	2.5 g
PBS without Ca^2+^ and Mg^2+^ Gibco, #10010-023	94.7%	49.85 mL
Triton X-100	0.3%	150 μL
Total	N/A	50 mL

Dissolve BSA completely before use. Store at 4°C for up to 2 weeks.

**Table:** Antibody dilution buffer (1% BSA, 0.3% Triton X-100 in PBS)

Reagent	Final concentration	Amount
BSA	1%	0.5 g
Triton X-100	0.3%	150 μL
PBS without Ca^2+^ and Mg^2+^ Gibco, #10010-023	98.7%	49.85 mL
Total	N/A	50 mL

Dissolve BSA completely before use. Store at 4°C for up to 2 weeks.

## Step-by-step method details

### pH treatment and fixation of BMDMs

**Timing: 2 days**

This section describes how to treat mature BMDMs with MGM at different pH levels and subsequently fix the cells for downstream immunofluorescence staining.

1. Plate BMDMs in gelatin-coated wells.
  a. On Day 7, detach BMDMs from the plate by adding 15 mL cold 2.5 mM EDTA (in 1x PBS) and incubate for 5–10 min at RT.
  b. Collect cells by pipetting, centrifuge at 500 × *g* for 5 min, and resuspend the cell pellet in fresh MGM.
  c. Plate 300K BMDMs per well into a 24-well plate containing gelatin-coated coverslips.

***Note:*** Plating density can alter cell morphology and size, influencing imaging and analytical accuracy. It is recommended to titrate the cell density for optimal imaging results.

2. Culture cells for at least 4 h.
  a. After cells become adherent, replace the medium with pH 7.4 MGM to remove debris and non-adherent or excessive cells.
  b. Culture macrophages overnight in regular MGM at pH 7.4, 37°C, 5% CO_2_.
3. pH treatment (day 2).
  a. Start the pH treatment by replacing the medium with MGM adjusted at pH 7.4, 6.5, or 6.0.
  b. Incubate the BMDMs for 4 h at 37°C, 5% CO_2_.

***Note:*** Make sure the medium is pre-warmed and equilibrated with 5% CO_2_ before use (at least 1 h at 37°C, 5% CO_2_). It is important to include a control condition with fresh pH 7.4 medium, because prolonged incubation may cause medium pH shift and medium change triggers mild stress response. We also recommend verifying the medium pH at the endpoint to ensure correct medium pH throughout the experiment.

4. Fixation.
  a. At desired time, wash cells gently once with pre-warmed 1×PBS.
  b. Fix cells with 500 μL 4% paraformaldehyde (PFA) per well for 15 min at RT.

**CRITICAL:** Dilute PFA stock with 1x PBS immediately before use.

5. Wash cells gently three times with 1x PBS to remove residual PFA.

Potential stopping point: Fixed samples can be stored in1x PBS at 4°C overnight, protected from light, before proceeding to immunofluorescence staining.

### Immunofluorescence detection of BRD4 condensates in BMDMs

**Timing: 2 days**

This section describes immunofluorescence detection of BRD4 condensate to assess pH sensitivity in BMDMs.

6. Permeabilization.
  a. Permeabilize cells by adding 500 μL of permeabilization buffer per well for 5 min at RT.
  b. Wash the cells three times with 1x PBS, 5 mins per wash.
7. Blocking.
  a. Incubate cells in 500 μL blocking buffer per well for 1 h at RT.
8. Primary antibody incubation.
  a. Dilute BRD4 primary antibody (1:100; final 5 ng/μL) or an isotype control IgG (final 5 ng/μL) in antibody dilution buffer.
  b. Add 250 μL of the antibody solution per well and incubate overnight at 4°C with gentle rocking.

***Note:*** BMDMs have a moderate expression of BRD4. Overnight incubation with the BRD4 antibody from Abcam yields optimal signal-to-noise ratio. We recommend including an isotype control in experiments to verify BRD4 staining, especially in cell types with low to moderate expression.

9. Secondary antibody incubation.
  a. Wash cells three times with 1x PBS, 5 mins per wash.
  b. Incubate with secondary antibodies diluted at 1:1000 in antibody dilution buffer for 1 h at RT in the dark with rocking. We recommend using secondary-only antibodies as a negative control in step 8 to assess non-specific secondary signal.

***Note:*** For confocal imaging, we used Alexa Fluor 488 or 647-conjugated anti-rabbit IgG to visualize the change of BRD4 condensates. For STED imaging, we used STAR RED-conjugated anti-rabbit IgG, which offers superior resolution (∼30 nm), photostability, and tolerance to high STED laser intensities.

10. Nuclear staining and mounting.
  a. Wash cells three times with 1xPBS, 5 mins per wash.
  b. Stain nuclei with Hoechst 33342 (1:1000 diluted in PBS) for 5 min at RT.
  c. Wash three times with 1xPBS, 5 mins per wash.
  d. Mount coverslips on glass slides using ProLong™ Glass Antifade Mountant.
  e. Store the sample at 4°C for at least 18 h until mounting medium cures.

***Optional:*** Seal slides with Fixo gum for extended storage.

Potential stopping point: Samples can be stored at 4°C for 3–5 days in the dark before imaging.

11. Confocal microscopy.
  a. Acquire Z-stack images using ZEISS 880 laser scanning confocal microscope with 63x/1.4 oil objective.
  b. Apply Airyscan processing for improved resolution and signal-to-noise ratio.

***Note:*** In Z. Wu et al., the following confocal imaging settings were applied: 1024 × 1024 pixels, 1.8× zoom factor, 16-bit pixel depth, 0.2 μm z-stack size across the entire samples. The laser power was set to 5 and master gain was 750.

12. STED microscopy.
  a. Acquire super-resolution images using a Nikon Eclipse Ti2-I/STED microscope with a 100x oil objective.
  b. Perform deconvolution using Huygens Professional (SVI) with the STED-specific point spread function (PSF) and the CMLE (Classic Maximum Likelihood Estimation) algorithm to improve resolution and structural fidelity (Figure 1B).

***Note:*** In Z. Wu et al., the following STED imaging settings were applied: 7 × 7 μm field; 40 nm pixel size, STAR RED dye (excitation 5.75%; STED 17%).

***Note:*** Although this protocol focuses on endpoint immunofluorescence, complementary live-cell imaging approaches can be used to capture temporal and dynamic axes of pH-dependent condensate regulation.^1^

### Image analysis and quantification of pH-dependent BRD4 condensates

This section describes the image analysis pipeline that quantify BRD4 condensates in BMDMs. CellProfiler is used to count the number of BRD4 condensates from confocal images. A custom Matlab program is used to quantify the size and intensities of BRD4 condensates from STED images.

### Image processing with Fiji

**Timing: 5 min per stack**

Here we describe the steps by which confocal images are processed in FIJI as TIF for subsequent image quantification.

13. Splitting of imaging files into separate channels and Z-frames.
  a. Load the code TIF_Macro.ijm (Data S1) into FIJI.
  b. Click “Run” in the bottom left corner of the editor page.
  c. Specify the “Input Directory” where the files to be processed by splitting are located.
  d. Specify the “Output directory” where the resulting split files will be saved.

***Note:*** The script is provided in the supplementary files and will process every image in that “Input directory” that has the specified file. The end of this script is that images Z-stacks will be projected and saved as TIF for input CellProfiler.

14. Image processing for example.
  a. Open the image file.
    i. dragging the file into FIJI.
  b. Generate a Z-projection.
    i. Select Image ->stack -> Z-project.
    ii. Choose Maximum Intensity and click OK.
      ***Note:*** This will create a new image window.
  c. Adjust display range.
    i. Go to Image -> adjust -> Brightness/Contrast.
    ii. Click set.
    iii. In the Set Display Range dialog, fill in the Minimum displayed value and Maximum displayed value based on the intensity values indicated by your cursor when hovering over background and condensate regions.
    iv. Click OK to apply.
      ***Note:*** The changes in BRD4 condensates can be observed between pH 7.4 and 6.5. Fewer and smaller BRD4 condensates are shown at pH 6.5 (Figures 1C and 1D).
  d. Add a scale bar.
    i. Select Analyze -> tools -> Scale Bar.
    ii. Specify length, color, and font size according to the image magnification (e.g., 2 μm).
    iii. Click OK to apply the scale bar.
  e. Save and export images.
    i. Export a display-quality image as PNG format.

### Quantifications of BRD4 condensates with CellProfiler

**Timing: ∼26 s per image**

CellProfiler^3^ is an open-source, general-purpose image analysis tool designed for developing reproducible and reusable pipelines to quantify large image datasets (https://cellprofiler.org/). Here we use CellProfiler to quantify the number of BRD4 puncta within nuclei using the ExampleSpeckles.cppipe pipeline available at https://cellprofiler.org/examples.

15. Prepare images for analysis.
  a. Rename the BRD4 image as BRD4_hrh2a×2.tif and the DAPI image as DAPI_hrhoe2.tif. Ensure that both files are saved as grayscale.tif format.

**CRITICAL:** Images must be saved as **.tif** format with grayscale for compatibility with CellProfiler.

**Figure 2:**
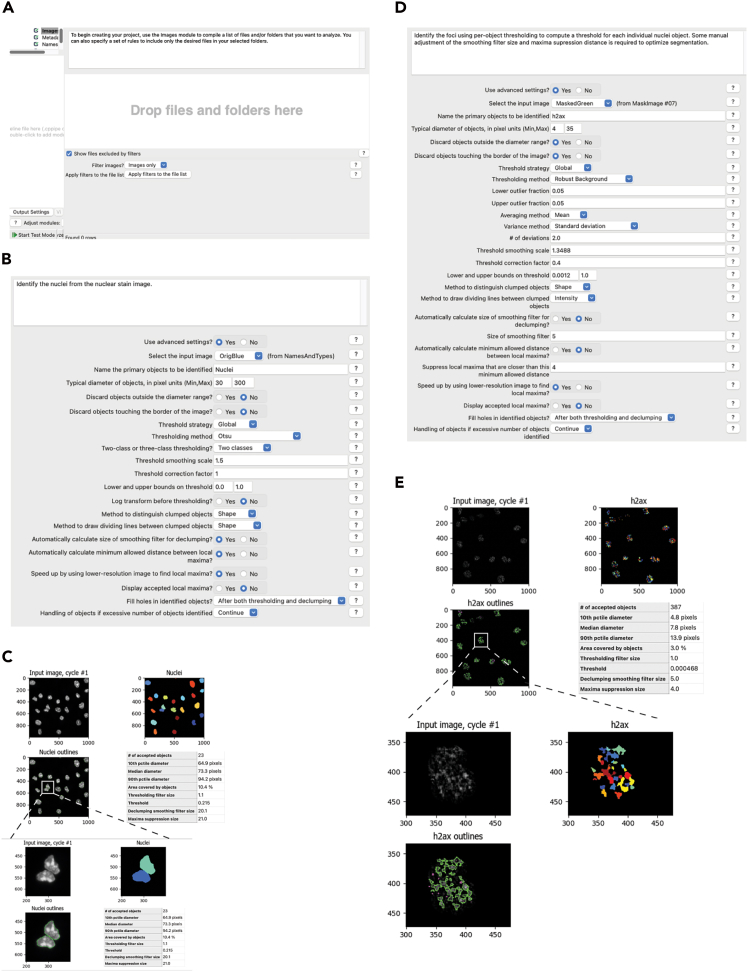
Image analysis in CellProfiler (A) Visualization of the Images module in the CellProfiler pipeline where individual files, groups of files, or folders can be dragged for processing. (B) Adjustment of parameters in the IdentificationPrimaryObjects module for nuclei detection. (C) Visualization of nuclei segmentation by clicking the Start Test Mode. (D) Adjustment of parameters in Identification PrimaryObjects module for BRD4 puncta detection. (E) Visualization of BRD4 puncta identification using Start Test Mode.
17. Load the analysis pipeline.
  a. Load the pipeline “ExampleSpeckles.cppipe” into the corresponding window.
18. Test the pipeline.
  a. Click Start test mode and then Step to execute the pipeline step-by-step.
19. Verify nuclei segmentation (Figures 2B and 2C).
  a. Measure the size of nuclei and visually check nuclei outlines accurately match the DAPI signal.
20. Verify BRD4 puncta identification (Figures 2D and 2E).
  a. Zoom in on a single cell and examine BRD4 puncta detection.
  b. Confirm that all puncta are identified and that their outlines align with the original BRD4 signals.

***Note:*** If puncta outlines are inaccurate or incomplete, adjust the object diameter and threshold parameters in IdentifyPrimaryObjects.

16. Load images into CellProfiler.
  a. Open CellProfiler.
  b. Drag and drop the BRD4_hrh2ax2.tif and DAPI_hrhoe2.tif images into the area labeled “Drop files and folders here”. (Figure 2A)

21. Adjust parameters.
  a. Modify the diameter of objects and threshold settings in IdentifyPrimaryObjects until BRD4 puncta are correctly identified.
  b. Once optimized, exit test mode and run the pipeline on all images for batch quantification.
22. Export and analyzed results.
  a. In ExportToSpreadsheet, Set the Output file location to your desired destination folder.
  b. After pipeline execution, three files will be generated: h2ax.csv, nuclei.csv, and image.csv.
  c. Open nuclei.csv to locate the key output columns:
    i. ObjectNumber: identifies individual nuclei).
    ii. Children_h2ax_Count: represents the number of BRD4 puncta (H2AX objects) associated with each nucleus.
    iii. Combine exported data using GraphPad Prism.

Quantify the number, area, and intensity of BRD4 puncta per image.

***Note:*** Additional spatial features (e.g., puncta radius/shape) can be quantified by customizing the Matlab analysis code once the puncta objectives are identified.

### Quantifications of BRD4 condensates using MATLAB

**Timing: ∼10 s per image**

A custom MATLAB program (Data S2) and its accompanying documentation (Document S1,) are provided as the supplementary files for quantitative analysis of BRD4 condensates from STED images. This program assumes that the user can call Bftools in the MATLAB path. Besides this there are a set of mandatory user inputs and optional user inputs. The program analyzes all STED image files that are present in a directory and outputs a PDF file of the contour graph and a CSV file containing the raw values each for cytosolic and nuclear condensates.

23. Open sted_analysis.m in MATLAB to run the program.
24. Modify the input directory in Line 14 of the program.
25. Adjust the pixel resolution in Line 15 of the program.
26. Confirm that the channels being analyzed correspond to those specified in Lines 37–38 of the program.
27. (Optional) Change output file names in Lines 26–29 of the program if needed.
28. (Optional) Customize Plot features of the contour graphs in Lines 110 (onwards) and 121(onwards).
29. After execution, the generated PDF plots can be further refined for presentation using any vector graphic software (e.g., Adobe Illustrator).

## Expected outcomes

This protocol provides detailed instructions for pH medium preparation and enables visualization and quantification of BRD4 condensates in BMDMs under different extracellular pH conditions. At pH 7.4, BRD4 typically forms bright puncta within the nucleus. Under acidic pH, the number and size of BRD4 puncta are expected to reduce significantly, reflecting the disruption of BRD4 condensates at an acidic intracellular pH. Quantification using FIJI and CellProfiler could help reveal the degree of reduction in condensate number, size and relative intensity under matched acquisition setting.

For higher-resolution imaging assessment, STED provides improved resolution (∼30–40 nm) to analyze individual BRD4 puncta and reveals detailed differences in their spatial organization across pH conditions. The provided MATLAB script outputs the distribution of puncta intensity and apparent size. Together, these pipelines provide a reproducible framework for assessing pH-dependent regulation of BRD4 condensates and can be adapted to assess other transcriptional condensates.

***Note:*** If stained puncta differ between conditions, the results should be interpreted with caution. Importantly, puncta intensity changes in STED images may not necessarily reflect changes in protein abundance. In fact, BRD4 protein level is found to be comparable between pH 7.4 and pH 6.5, while BRD4 condensates significantly differ. It is important to know that the diameter of identified puncta may not precisely reflect the size of condensates due to technical limitations, such as the size of bound antibodies.

## Quantification and statistical analysis

All image data are processed and analyzed using FIJI, CellProfiler, and MATLAB as described in the corresponding sections. For quantification, confocal or STED images are first preprocessed in FIJI to adjust contrast, remove background, and project Z-stacks into 2D images. BRD4 puncta were then segmented and quantified in CellProfiler using a customized pipeline based on ExampleSpeckles.cppipe. The pipeline identified nuclei from DAPI staining and BRD4 puncta as primary objects, linking puncta to their corresponding nuclei. The output parameters included the number, area, and intensity of BRD4 puncta per nucleus.

Data from at least three biological replicates and a minimum of 60 nuclei per condition are included in the analysis. Images exhibiting poor focus, uneven illumination, or incomplete nuclei segmentation are excluded.

All numerical results are exported from CellProfiler as.csv files and analyzed using GraphPad Prism. Comparisons between two groups (e.g., pH 7.4 vs. pH 6.5) were performed using unpaired two-tailed Student’s *t*-tests. Data are presented as mean ± SEM, and significance thresholds were set as *p* < 0.05 (∗), *p* < 0.01 (∗∗), and *p* < 0.001 (∗∗∗). Example data files and analysis scripts are provided as supplementary datasets to facilitate reproducibility.

## Limitations

The protocol and conditions described here are optimized for murine BMDMs. Studies of tissue resident macrophages, other immune and non-immune cell types, or cells from other species will likely require empirical optimization for the pH range, exposure duration, fixation/permeabilization conditions, and imaging/acquisition parameters. It is important to know that the sensitivity of BRD4 condensates to extracellular acidic pH varies among cell types. This may rise from difference in pH buffering capacity or composition of transcriptional condensates among cell types.

In addition, medium pH adjustment may introduce small ionic and osmolarity differences. Although not included in this protocol, additional control medium with matched ionic strength can be further considered to exclude potential impact by different Na^+^, Cl^-^ or HCO_3_^-^.

Maintaining stable pH in culture medium may present technical challenges. Different cell types have distinct metabolic activities and often require specific basal culture media and supplements, which can alter buffering capacity and pH drift over time. Moreover, different medium buffer capacities, availabilities of non-bicarbonate commercial medium, may limit direct translation of the described protocol to certain studies.

## Troubleshooting

### Problem 1

BRD4 condensates do not respond to acidic pH (related to steps 3 in the step-by-step method details).

### Potential solution

First, vary medium pH calibration. Improper pH adjustment is a common cause of this issue. Always confirm the pH of the media immediately before use and ensure equilibration in a 5% CO_2_ incubator for at least 1 h prior to measuring pH. CO_2_ exchange can shift the final pH by 0.1–0.3 units, and media stored for several days may experience additional drift.

Second, ensure macrophages are naive prior to pH treatment. Activated macrophages undergo metabolic and ion-transport remodeling, making their intracellular pH less insensitive to changes in the extracellular pH. Avoid any contaminations when preparing the BMs.

Third, sample multiple imaging fields. BMDMs exhibit intrinsic heterogeneity in pH sensing. Acquire images from multiple, randomly selected fields to obtain unbiased visualization and quantification of BRD4 condensates.

### Problem 2

Some BMDMs are sensitive to acidic pH, whereas others are not (related to step 14 in the step-by-step method details).

### Potential solution

Heterogeneous responses among BMDMs can arise from variable confluency, differentiation states, or technical inconsistency. Maintain moderate confluency (60–70%) and ensure cells are uniformly differentiated and attached before pH treatment. During imaging, acquire images with standardized Z-stack and Airyscan settings to account for nuclear height variation and condensate visibility.

### Problem 3

The default settings do not apply to the data when analyzed using CellProfiler (related to steps 15–22 in the step-by-step method details).

### Potential solution

Re-examine the identifyPrimaryObjects settings in the CellProfiler pipeline. The diameter of BRD4 condensates and the threshold parameters are critical for accurate detection. If the diameter range is too broad or the threshold is too low, smaller or dimmer puncta may be included, masking pH-dependent differences.

Optimize these parameters using Test Mode and visually confirm that all puncta are correctly identified. Ensure consistent illumination and exposure across all samples, as uneven imaging can also obscure true differences.

## Resource availability

### Lead contact

Further information and requests for resources and reagents should be directed to and will be fulfilled by the lead contact, Dr. Xu Zhou (xu.zhou@childrens.harvard.edu).

### Technical contact

Technical questions related to the execution of this protocol should be directed to and will be answered by Dr. Zhongyang Wu (wu.zhongyang@childrens.harvard.edu).

### Materials availability

This study didn’t generate new unique reagents.

### Data and code availability

• The condensates identification pipeline used for quantifying confocal microscopy data was downloaded from the CellProfiler website (https://cellprofiler.org/examples).
• All original code is available in this paper’s supplementary information.

## Acknowledgments

The experiments reported in this paper were performed in collaboration with the Center for Open Bioimage Analysis (COBA) which is supported by 10.13039/100000057National Institute of General Medical Sciences 10.13039/100000002NIH P41, GM135019. This work was supported by 10.13039/100000002NIH RC2DK122532 and R35GM142683 to J.R.T, 10.13039/100000002NIH R35GM151000, P30DK034854, 10.13039/100009576Kenneth Rainin Foundation and 10.13039/100001341Smith Family Foundation to X.Z.

## Author contributions

Z.W. and X.Z. conceived the project. Z.W. and Z.Z. optimized the pH treatment protocol. Z.W. and R.K. developed the image analysis pipeline. Z.W. and X.Z. wrote the original draft of the manuscript. All authors read and revised the manuscript.

## Declaration of interests

The authors declare no competing interests.

## Supplemental information

Document S1. Documentation for the custom Matlab code for STED image analysis, related to step 23–29 in the step-by-step method details

Data S1. FIJI macro code for generating TIF files, related to step 13 in the step-by-step method details

Data S2. Matlab program for STED images analysis, related to step 23 in the step-by-step method details
