# Supplementary material for: Protocol for immunofluorescence detection and quantitative analysis of pH-dependent transcriptional condensates
Source: STAR Protoc. 2026 Mar 11;7(1):104425. doi: 10.1016/j.xpro.2026.104425 (PMC12994006; doi:10.1016/j.xpro.2026.104425)
Supplement: Document S1. Documentation for the custom Matlab code for STED image analysis, related to step 23–29 in the step-by-step method details [file mmc1.pdf]

Table of Contents

PRECHECKS 1
MANDATORY USER INPUTS 1
OPTIONAL USER INPUTS 1
FILE OPENING AND EXTRACTION OF REQUIRED CHANNEL 2
USER CHECK FOR WHICH IMAGE CHANNELS TO USE 2
THE ACTUAL ANALYSIS PART 2
WRITING TO TABLE 3
GENERATING AND PRINTING THE CONTOUR 4
OPTIONAL USER INPUT TO CHANGE NUCLEI CONTOUR PLOT FEATURES 4
OPTIONAL USER INPUT TO CHANGE CYTOSOLIC CONTOUR PLOT FEATURES 5

PRECHECKS

```
% First thing to make sure is that you have the latest bftools in your
% path. You can download this from the OpenMicroscopy website
%  https://www.openmicroscopy.org/bio-formats/downloads/

% Clear any existing variables in the workspace
clear all
```

MANDATORY USER INPUTS

Find all files in the current directory matching the pattern "*.obf" We typically run the different conditions separately*

```
dir1 = dir("*.obf");
RES=25; % This here is the resolution per pixel.

% Loop through each STED image file in the directory
for i = 1:size(dir1)

    % Get the name of the file that is being processed. The semicolon is
    % purposely left out to print on screen the name of the file that is
    % being processed
    iname = dir1(i).name
```

```
iname =

    'Copy_of_IMG0014_pH7.4_5.obf'


iname =

    'IMG0014_pH7.4_5.obf'
```

OPTIONAL USER INPUTS

Change these for any required changes to output file names

```
out_name_out = strcat(iname, '_out', '.pdf');
out_name_in = strcat(iname, '_in', '.pdf');
out_csv_in = strcat(iname, '_in', '.csv');
out_csv_out = strcat(iname, '_out', '.csv');
```

FILE OPENING AND EXTRACTION OF REQUIRED CHANNEL

Opening the above image file

```
img1 = bfopen(iname);
```

```
Reading series #1
    .
Reading series #2
    .
Reading series #3
    .
Reading series #4
    .

Reading series #1
    .
Reading series #2
    .
Reading series #3
    .
Reading series #4
    .
```

USER CHECK FOR WHICH IMAGE CHANNELS TO USE

Extract the red and blue channel images. In our case it was the first and third channel. Change this for the channel order

```
img1_red = img1{1,1}{1};
img1_blue = img1{3,1}{1};
```

THE ACTUAL ANALYSIS PART

First we apply multilevel thresholding to the Nuclei channel and isoalte the thresholded image

```
level_blue = multithresh(double(img1_blue), 3);
BW_t_blue = imquantize(img1_blue, level_blue);
BW_t_blue = (BW_t_blue - 1);
BW_t_blue = max(BW_t_blue, 0);
BW_blue = logical(BW_t_blue);

% Next we pre-process the condensate channel
img1_red3 = wiener2(img1_red, [5 5]); % Wiener filter for noise reduction
```

```
    background = imopen(img1_red3, strel('disk', 4)); % Estimation of
background
    img1_red2 = imsubtract(img1_red3, background); % Background subtraction

    % We isolate the areas inside the nuclei. Change the number 4 below if
    % your signal to noise is not that great.
    img1_red_inside = img1_red2 .* uint16(BW_blue);
    level = multithresh(double(img1_red_inside), 4);
    BW_t = imquantize(img1_red_inside, level);
    BW_t = (BW_t - 1);
    BW_t = max(BW_t, 0);
    BW = logical(BW_t);

    % We filter the areas that are between two size threshold to prevent
    % aggregates and really small speckles. Change this if you change the
    % magnification of the microscope.
    CC = bwpropfilt(BW, "Area", [3 80]);

    % Of the isolated regions, we extract region properties and store them in
a table
    myrois = regionprops(CC, img1_red,
{'Centroid', 'MeanIntensity', 'EquivDiameter', 'Area'});
    myroi_table = struct2table(myrois);

    % Concatenate tables appending the current image to other images in the
    % directory
    if i == 1
        T = myroi_table;
    else
        T = [T; myroi_table];
    end
     % We repeat the exact code from above but for the cytosol.
    img1_red_outside = img1_red2 .* uint16(~BW_blue);
    level_out = multithresh(double(img1_red_outside), 4);
    BW_t_out = imquantize(img1_red_outside, level_out);
    BW_t_out = (BW_t_out - 1);
    BW_t_out = max(BW_t_out, 0);
    BW_out = logical(BW_t_out);
    CC_out = bwpropfilt(BW_out, "Area", [3 80]);
    myrois_out = regionprops(CC_out, img1_red,
{'Centroid', 'MeanIntensity', 'EquivDiameter', 'Area'});
    myroi_table_out = struct2table(myrois_out);
    if i == 1
        T_out = myroi_table_out;
    else
        T_out = [T_out; myroi_table_out];
    end

end
```

WRITING TO TABLE

```
    writetable(T,  out_csv_in);
```

```
    writetable(T_out,  out_csv_out);
```

GENERATING AND PRINTING THE CONTOUR

Finally we generate histograms and contour plots for EquivDiameter and MeanIntensity This adjusts the binning regions. Modify it based on your data

```
Xedges = 70:10:400;
Yedges = 0:2:40;
% RES here is the pixel size.
% this can be changed based on the mciroscope
T_out.EquivDiameter = T_out.EquivDiameter * RES;
T.EquivDiameter = T.EquivDiameter * RES;
z = histcounts2(T.EquivDiameter, T.MeanIntensity, Xedges, Yedges);
```

OPTIONAL USER INPUT TO CHANGE NUCLEI CONTOUR PLOT FEATURES

```
figure(1)
contourf(Xedges(1:size(z,1)), Yedges(1:size(z,2)), z.', 'ShowText', 'off')
colormap(flipud(hot))
% Change these for any required changes to axis scaling
axis([70 200 0 38])
xlabel('Diameter (nm)', 'FontSize', 12)
ylabel('Mean Intensity', 'FontSize', 12)
saveas(gcf,out_name_in)
```

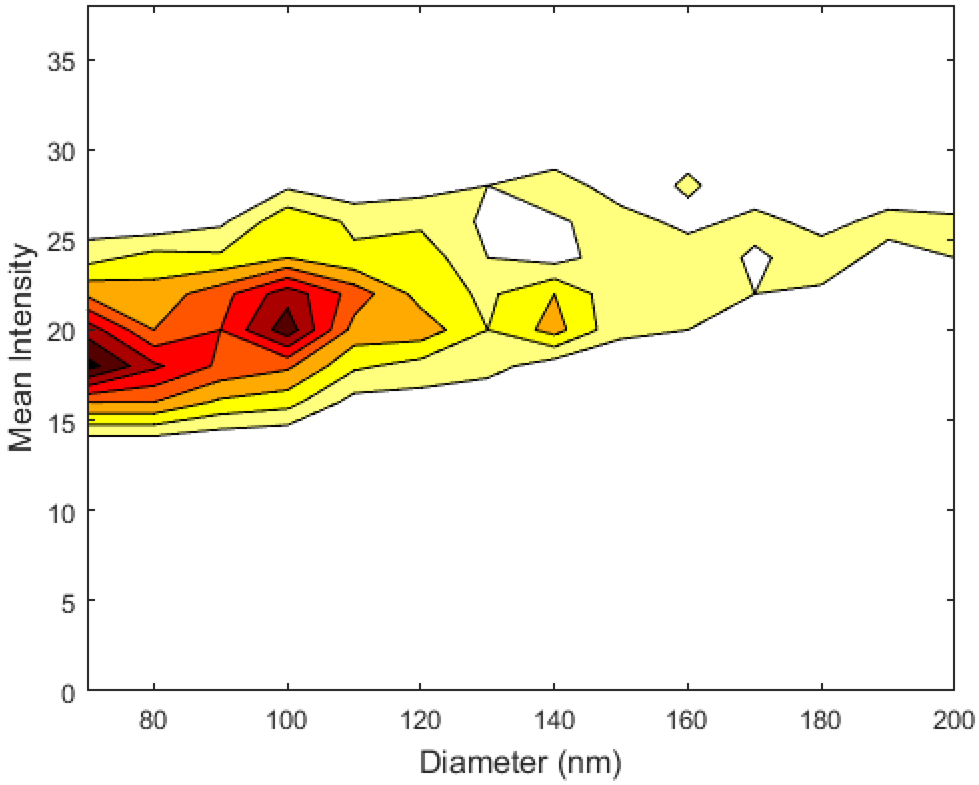

OPTIONAL USER INPUT TO CHANGE CYTOSOLIC CONTOUR PLOT FEATURES

```
z1 = histcounts2(T_out.EquivDiameter, T_out.MeanIntensity, Xedges, Yedges);
figure(2)
contourf(Xedges(1:size(z1,1)), Yedges(1:size(z1,2)), z1.', 'ShowText', 'off')
colormap(flipud(hot))
% Change these for any required changes to axis scaling
axis([70 200 0 38])
xlabel('Diameter (nm)', 'FontSize', 12)
ylabel('Mean Intensity', 'FontSize', 12)
saveas(gcf,out_name_out)
```

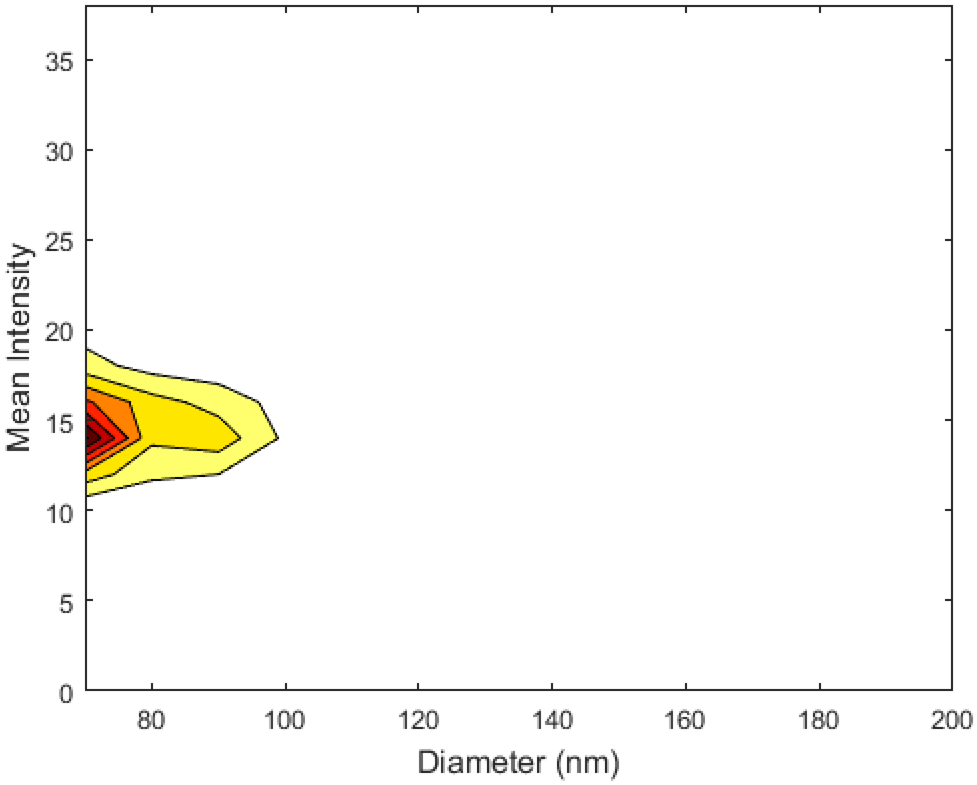

Published with MATLAB® R2023a
